# The Study of Geographical Distribution in the Analysis of Domestication as an Evolutionary Process: Tensions in Alphonse de Candolle’s Approach

**DOI:** 10.1007/s10739-024-09785-5

**Published:** 2024-09-23

**Authors:** Miriam Álvarez-Tostado, Alfredo Bueno-Hernández, Ana Barahona, Fabiola Juárez-Barrera

**Affiliations:** 1 Programa de Posgrado en Ciencias Biológicas, Coordinación de Estudios de Posgrado, Universidad Nacional Autónoma de México (UNAM), Mexico City, 04510 Mexico; 2 Facultad de Estudios Superiores Zaragoza, Universidad Nacional Autónoma de México (UNAM), Mexico City, 09230 Mexico; 3 Facultad de Ciencias, Universidad Nacional Autónoma de México (UNAM), Mexico City, Mexico

**Keywords:** Domestication, Alphonse de Candolle, Darwin, Geographical distribution, Biogeography, Artificial selection

## Abstract

Interest in the study of domesticated plants increased near the end of the 18th century, mainly because of their economic potential. In the 19th century, there was a new focus on the historical understanding of species, their origin, changes in their distribution, and their evolutionary history. Charles Darwin developed an extended interpretation of species domestication, considering variations, reproduction, inheritance, and modification as standard processes between wild and domesticated organisms. In this context, one relatively neglected aspect was the geographical distribution of domesticated species. Alphonse de Candolle addressed and developed in detail the question of the geographical origin of cultivated plants. Since 1836 Alphonse de Candolle had been studying the topic and obtained evidence that contributed to understanding aspects such as the center of origin, dispersion, competition, selection, and time of domestication. Although Darwin himself admitted that *Géographie botanique raisonnée* (de Candolle, Alphonse,de. Géographie botanique raisonnée; ou, exposition des faits principaux et des lois concernant la distribution géographique des plantes de l’epoque actuelle, 2ème tome. Paris: Masson.) was of great help to him in the development of his evolutionary theory, the importance of de Candolle’s contribution is seldom recognized. Our purpose is to detail the dialogue between Alphonse de Candolle and Darwin on the geography of domesticated plants, to understand some of the most critical discussions that contributed to the reinterpretation of domestication under the Darwinian proposal of modified descent.

## Introduction

Even before they began to have an intellectual relationship, Darwin and de Candolle shared a common interest. Since his voyage on the HMS *Beagle*, Darwin was struck by the potato he had found in the Chonos Archipelago, and he wondered if this remote land might have been the birthplace of the common potato (Darwin 1839, pp. 347–348). Before the publication of Charles Darwin’s *Variation of Animals and Plants Under Domestication* (1868), domestication was interpreted more as a resource management issue than an evolutionary process. Precisely one of the most valuable contributions of the analysis developed in *Variation* (1868) was that Darwin, based on plentiful empirical and experimental evidence, was able to lay out the thesis that the management techniques practiced in organisms under domestication (artificial selection, hybridization, crossing, and interbreeding) represented a model of biological phenomena that also occurred within wild populations. One of his main aims in this work was to extend and support with further information his first chapter of the *Origin of Species* (Darwin 1859) as well as to explain the origin of the observed variation and how it is inherited, taking domesticated organisms as a model to argue that, under natural conditions, it is foreseeable that the variation is much greater (Brown 2010, p. 2523). He managed to go beyond this goal and not only reveal the origin and importance of variations, but also pose questions about inheritance and the patterns observed in the intergenerational transmission of characters. Likewise, he linked the reproduction and ontogenetic development processes to the explanatory domestication framework, analogous to species modification. That is, Darwin managed to unify various biological processes in the domestication model to argue that if these elements allow us to understand changes (morphological, physiological, and behavioral) in a limited context (under human control), then it was highly probable that, in a natural context where variation, reproduction, development, and inheritance are also concatenated (in an indeterminate succession) the results would be more profound and the initial phases of the generation of new species could be observed.

Undoubtedly, the Darwinian interpretation expanded the meaning of domestication since the extrapolation in time of the processes that occurred in domestication could explain the origin of new species. This conceptual unification by Darwin stimulated the response of several authors around the evidence that existed on domestication as an evolutionary event. Here, we will address the case of Alphonse de Candolle. It has been said that de Candolle did not support evolutionary thought until he read the copy of the *Origin of Species* sent to him by Darwin shortly after its publication (Egerton 2019, p. 7). We propose that his transition was not so simple. From a more detailed examination of his three books on plant domestication (1835, 1855, and 1883) and his correspondence with Darwin, it can be seen that de Candolle initially raised severe objections to the Darwinian thesis. De Candolle did not admit at the outset that the variation observed in cultivated plants could be extrapolated to wild plants and, therefore, it could not explain the emergence of new species. However, as the debate on natural selection expanded to discussions of inheritance and modification, and as he corresponded with Darwin, there was a shift in his thinking, as demonstrated in his texts published after 1862. Finally, in *Origine des plantes cultiveés* (1883), de Candolle adopted the Darwinian theory and integrated the spatial distribution component into the evolutionary interpretation of domestication.

## First Considerations in the Study of Domesticated Plants

Alphonse de Candolle’s extensive work on domesticated plants is part of a broader context. Since the mid-18th century, there had been an interest in knowing the origin of cultivated species. Linnaeus argued that, in principle, cultivated plants arose from human intervention in wild species. In many cases, the wild species from which they were derived were unknown, and due to this lack of knowledge, he did not include information on their geographical distribution (Wijnands 1986, p. 67). In this way, study of the distribution of cultivated plants remained a task calling for further work. It has been pointed out that in Linnaeus’ time, living conditions were harsh, and there was a more direct dependence on animals, plants, and natural materials for food, clothing, shelter, and goods. Any failure in humanity’s relationship with nature could have dire, if not fatal, consequences. Hence, the recognition, naming, and classification of different types of plants was, for Linnaeus, more of a pragmatic than an abstract matter. Linnaeus showed in his writings a particular concern for the technological underdevelopment that caused crop losses, with unfortunate economic and human consequences (Reid 2009, p. 25). He promoted the centralist management of natural resources by the state that would boost the Swedish economy. Linnaeus believed that natural history played a prominent role in economics (Koerner 1999). He looked for substitutes for luxury goods such as coffee, tobacco, tea, and silk so that Sweden would not have to import them (Müller-Wille and Charmantier 2012, p. 6).

Thomas Malthus also shared an interest in agricultural production. From the first edition of his well known *Essay on the Principle of Population* in 1798 to its condensed version in 1830, he focused on how agricultural production could be increased (Sinha 1999, p. 1528), and he reached the conclusion that agricultural improvement would be insufficient for sustaining growing human demands. In contrast to this utilitarian interest, Alphonse de Candolle had a more strictly intellectual interest (Smith 1969, p. 2). In works such as the *Géographie botanique raisoneé* (1855) and *Origine des plantes cultiveés* (1883), he did not deal with the subject of agricultural production or its economic repercussions as his main focus. In the preface of his *Origine*, he specified that his work appealed to the interests of botanists, farmers, historians, philosophers, and even travelers, but did not allude to economists. He did not establish that his study was expressly intended to serve to satisfy the human need for food. Instead, its purpose was to correct Linnaeus’s deficient information about the distribution and geographical origin of cultivated plants, since about three-quarters of Linnaeus’s indications of the location of cultivated plants were incomplete or erroneous.

Other authors as well addressed domesticated organisms and their relationship with their geographical space in several publications, since the end of the 18th century. For example, in *Histoire naturelle de la France méridionale* (1783), Abbot Giraud Soulavie examined the topography of plants between the Mediterranean and the summit of the Cévennes. Soulavie distinguished five vegetation zones staggered in barometric order, each characterized by a dominant crop; the first with orange plantations, then olive, the next level with vines and mulberry trees, then chestnut trees, and on the upper level, where cultivation was no longer possible, the vegetation was represented by fir and alpine plants. Soulavie’s work is considered one of the first biogeographic profiles that represent the variation of climate and vegetation as a function of altitude (Gómez-Gutiérrez 2022, p. 440).

Some years later, the English farmer Arthur Young made several trips to Great Britain, Ireland, and France, intending to observe the agricultural systems in each country and evaluate successful models (Riches 1967, p. 6). In the case of France, Young (1792) concluded that the diversity of climates is associated with the geographical location and terrain of the land. He was the first to notice the limits of cultivation crossed going from north to south or south to north, such as olive, mulberry, corn, and vine. He also presented a map of the different arable soils of France (Allen and Ó Gráda 1988, p. 112). Several decades later, the French botanist Charles F. Martins, who was director of the Montpellier Botanical Garden (1851–1879), considered that thanks to Young’s research, agricultural geography had been created, which is nothing other than the biogeography of cultivated species (Martins 1856, p. 462).

In the early 19th century, Alexander von Humboldt brought plant geography to prominence with his study *Essai sur la géographie des plantes* (Egerton 2019, p. 1). The Prussian naturalist questioned the origin of cultivated species by stating the following:

The origin and the first homeland of the most useful plants to man, where they would be living with him since the earliest times, is as impenetrable a secret as that of the land of origin of domestic animals… [w]e need to find out in which region the cereals spontaneously arose, such as wheat, barley, oats, and rye. Plants constituting the native wealth of all the tropics’ inhabitants—the banana tree, *Carica papaya*, *Jatropha manihot*, and maize—have never been found in a wild state. (Humboldt and Bonpland 2009, p. 72)

This research agenda, carried out by several naturalists in the late 18th century and 19th century, contributed to the emergence of disciplines such as botanical and zoological geography. In these studies, the interpretations were often historical but far from evolutionary (Acot 1983, p. 43).

A pupil of Humboldt, the Danish botanist Joakim F. Schouw published a work on plant geography, *Grundzuge einer allgemeinen Pflanzengeographie* (1823), accompanied by an atlas, which showed a regionalization of areas based on climatic zones, dominant vegetation type and latitudinal lines (Rupke and Wonder 2000, p. 166; Ebach 2015, p. 53). Decades later, Martins (1856) reviewed the advances in botanical geography, which included the contributions of another work by Schouw, *Die Erde*,* die Pflanzen und der Mensch* (1851), in which he evaluated the influence that temperature exerts on vegetation, restricting most species to a specific range with polar and tropical boundaries. Schouw drew these opposite boundaries on a map of Europe and carefully plotted the boundaries of wild and cultivated plants and how they related to isothermal lines.

The cultivated plants that stand out the most in Schouw’s characterization were cereals, or bread plants, due to the food culture that had historically developed from these species. The established geographical boundaries of crops were so crucial to Schouw that he gave the name of “bread-line” to the northern boundary of the areas where cereals were planted. This strip did not represent a trend parallel to the circles of latitude but displayed curvatures towards the pole and the equator (Schouw 1852, pp. 130–131).

Another figure in 19th century botany who was a fundamental antecedent to Alphonse de Candolle in the question of botanical (and agricultural) geography and who also considered cultivated plants in his explanations of the geographical distribution of species was his father, Augustin Pyrame de Candolle. The elder de Candolle established himself as an expert in European botany, contributing numerous works that addressed the geographical distribution of plants as a scientific question. Cultivated plants were not the main topic in all these publications, however. Augustin de Candolle frequently considered the case of these species to exemplify aspects such as morphological variation, the response to environmental changes, or the naturalization capacity of the plants. That is, cultivated plants represented general phenomena observable in wild plants, including distribution patterns related to physical conditions.

Augustin de Candolle developed his career as a botanist while the evolutionary debate began to spread in France. In 1805, he published the third edition of the *Flore française* in collaboration with Jean-Baptiste Lamarck, in which they included a biogeographic map that represented the floristic provinces of the French territory (Ebach and Goujet 2006, p. 764). Some boundaries that were included in that map were the main crop areas, such as corn, olive trees, and grapes (Güttler 2015, p. 32). His direct relationship with Lamarck exposed him to ideas about the transformation of species through progressive change, from the simplest to the most complex forms, through the influence that environmental changes^1^ exerted on the use and disuse of anatomical structures (Gissis 2010, p. 217). Apart from the Lamarckian proposal, Augustin de Candolle met the Swiss naturalist Alexander Moritzi and his inclination for transformist explanations which he published in *Réflexions sur l’espèce en histoire naturellle* in 1842, a work in which he criticized the idea of species as created and fixed entities, presented his rejection of artificial taxonomic systems (such as the Linnaean), and raised arguments defending the environmental modification of species. He did this, however, without clearly explaining any process that generated the change. Among the most notable arguments that Moritzi considered in his analysis were the fossil record and domesticated species. Augustin de Candolle worked with Moritzi in the Conservatoire Botanique of Geneva between 1832 and 1836, and they had an exchange of letters in which Moritzi shared fragments of *Réflexions* with him before publishing it (Friedman and Endress 2020, p. 4).^2^

Despite the elder de Candolle’s exposure to early evolutionary thought, he held a fixist notion of species and appears to have spoken out moderately against transformism (Gassó and Eulàlia 2008, p. 695; Friedman and Endress 2020, p. 30), particularly the Lamarckian interpretation (Galera 2016, p. 5). Despite this, variation was a primary element in his theory of stations and habitations, as well as the idea of “war” or “struggle” between plants for resources such as light, humidity, nutrients, or space (La Vergata 2023, p. 226). The variation and competition could be observed in both native and domesticated plants, and allowed general explanations for the geographical distribution of plant species to be formulated:

All the plants in a country, within a particular shared place, are in a state of war. All are equipped with more or less efficient means of reproduction and nutrition. The first, which establish themselves by chance in a given locality, tend to exclude other species by occupying their same space: the largest suffocate the smallest; the most perennial replace those whose duration is shorter; the most fertile gradually take over the space that could be occupied by those who multiply more difficultly… When these limits are very close, the plant is more delicate; it can only live in a small number of localities, and for the same reason cannot naturalize far away or be easily cultivated… The wider these limits are, the more robust the plant is; the more it can live in diverse locations; in addition, it is also easy to cultivate and naturalize in the field. (Augustin de Candolle 1820, pp. 384–385)^3^

The junior de Candolle’s interest in botany, his training in that discipline, the considerations of variability, competition, and the environment for the development of sciences such as taxonomy and botanical geography, and the subsequent integration of this knowledge into the evolutionary debate towards the second half of the 19th century, are elements that demonstrate the significant influence on the study of domesticated plants that the entire work of his own father, Augustin P. de Candolle, exerted.

Although Alphonse de Candolle’s major influence was his own father (de Candolle 1862) he had the good fortune to read the works of Humboldt, Geoffroy Saint-Hilaire, and Jussieu, and to live with the staff of the Paris Museum and its visitors, which gave him access to the works and lectures of Lamarck, Cuvier, and Humboldt. Despite these many antecedents in botanical geography, which also referred to domesticated plants, some works have stated that the scientific study of the origin of cultivated species began with the work of Alphonse de Candolle, in his *Géographie botanique raisonné* (1855) (Hui-Li 1970, p. 3; Abbo, et al. 2022, p. 188). However, before that text, the young de Candolle had already addressed this question in *Distribution géographique des plantes alimentaires* (1836). In both works, he took a broad approach to the subject, including not only botanical but also archaeological, historical, and linguistic data in order to locate the center of origin of the cultivated plants.

A central problem in the study of domesticated plants was to know their spatial distribution, as this could shed light on their geographical origin, their diversification and the success of their cultivation outside their original area. Unlike other naturalists, such as Swainson (1835), who considered species and their ranges as fixed, de Candolle expressed his doubts on this issue. Already in the preface to his *Géographie botanique*, de Candolle wondered whether their primary distribution could be known from the present one and whether the changes in the organization of living beings have occurred by modifications of their predecessors or by the creation of new forms. Since then, he clearly approached his research with the historical dimension in mind (de Candolle 1855, pp. XI-XIII).

*Origine des plantes cultivées* (1883) represented an interpretation developed when the debate about Darwin’s theory of evolution had already spread throughout Europe and the US.^4^ Alphonse de Candolle discussed the question of the sites of origin of cultivated plants, at least since 1836, in the work *Distribution géographique des plantes alimentaires.* Later, in his most recognized text, *Géographie botanique raisonnée* (1855), he addressed the geographical origin of some domesticated forms once more. As can be seen, de Candolle had a long and persistent interest in documenting the relationship between cultivated plants and their spatial distribution, even before the publication of *On the Origin of Species* (1859) and his conversion to evolutionism.

### The Cultivated Plants Before, During, and After Meeting Darwin

In his 1836 work, *Distribution géographique des plantes alimentaires*, de Candolle reviewed only 23 species of food plants (1836, p. 60). The first geographical categorization was made, distinguishing “Old World plants” and “New World plants.” In this preliminary approach, he found a greater diversity of plants cultivated in Eurasia, spanning over 18 species. Eight of the total plants belonged to the grass family (barley, rye, oats, wheat, spelt, rice, millet, and maize).

This first work devoted to cultivated plants was one of the first spaces in which de Candolle aimed to investigate the place of domesticated species in discussions of botanical geography. He began by asserting that in both wild (spontaneous) and cultivated plants, the general patterns of spatial distribution are the same, because physical and physiological laws determine the distribution of any species. If the environmental conditions did not present significant changes, then the areas occupied by the organisms would be more or less stable. If a species were to leave the limits of its geographic distribution, it was highly likely that new conditions or competition with native organisms of a new area would stop its establishment. He wrote:

In this struggle of new species against the old ones, the place becomes occupied, as in many other fights, by the first occupant with the most numerous army. In their natural state, the species we cultivate are subject to the same laws. (de Candolle 1836, p. 4)

It is striking that de Candolle points out the competition between native organisms and introduced organisms, mentioning “the struggle” that is triggered by resources (including physical space) and affirming that the favored species will be composed by more individuals. In the *Géographie botanique* (1855), de Candolle admitted that in some cases new specific forms might derive from the old ones, and that most species date from earlier epochs and were subjected to geographical or climatic changes (Baehni 1955, p. 111). According to the epistolary evidence we know that de Candolle did not support evolutionary thinking even after reading *On the Origin of Species* (1859),^5^, contrary to what Egerton claims (2019). In 1862, de Candolle stopped categorically denying the modification of species, but he still doubted the participation of natural selection. However, we agree with Egerton that even in this early work, de Candolle already included elements that would later be present in the Darwinian framework. From an early period, a tension between fixist and transformist conceptions of the origin of species was developing in de Candolle’s thought. Although de Candolle agreed that the general processes that explain the distribution of wild plants are also applicable to plants that have been domesticated, it was necessary to examine an essential difference since cultivated species have been subject to a set of conditions related to the forms of management exercised by human beings.

The distribution areas occupied by cultivated plants showed significant differences compared to the areas of their wild relatives. The history of the respective distributions was different for each case. In wild plants the limits could be seen as natural, and it was highly probable that these distribution limits had been more or less stable for an indeterminate period (approximately centuries). On the other hand, the distribution of cultivated plants had no stable limits and could extend indefinitely (de Candolle 1836, p. 5).

However, although the spatial limits of cultivated forms could be so extensive due to their relationship to human needs such as religion, industry, commerce, and even aesthetic appreciation, de Candolle clarified that agricultural limits generally tend to be restricted based on various parameters, mainly in the case of native plants, because variables such as temperature, light, humidity, etc. are aspects that could hardly be controlled or modified as desired. What a farmer could certainly control was the selection and favoring of specimens that showed resistance to physical variables, which could ultimately increase the distribution of cultivated species: “Undoubtedly, with care, of course, and a judicious choice of the varieties he cultivates, he could compensate for certain climate inconveniences and thus advance some limits of cultivation” (de Candolle 1836, p. 6). Of course, this explanation put forward by de Candolle makes sense when considering how economic systems had influenced the introduction and naturalization of multiple introduced and domesticated species in regions far removed from their areas of origin (Anderson 1992, p. 141).

As can be seen, at a very early moment in Alphonse de Candolle’s career, notions such as “struggle,” “choice,” and “varieties” appear as elements of his own thought, although now inevitably associated with Darwinian theory. Indeed, they are key components of Darwin’s theories, but before the English naturalist integrated them into his vision, these aspects were already discussed within various fields of natural history. The idea of balance or equilibrium in nature was another concept used and scrutinized by many authors prior to Darwinism. The perpetual struggle between organisms was necessary to maintain this balance, and in fact, this struggle or competition could explain the limits of species’ geographical distribution (La Vergata 2023, p. 8). Alphonse’s father, Augustin de Candolle, was one of the first naturalists in the 19th century who realized the importance of investigating the relationships between organisms and their environment. In other words, he understood the relationship between the variations of individuals and the potential advantage that one trait or another can provide for survival in specific contexts. It is pertinent to note that in 1809, Augustin de Candolle had access to the French translation of *Essay on the Principle of Population* (Drouin 1994) and in 1816, on a trip to England, he was able to meet Thomas Malthus (de Candolle 1862, p. 265).^6^

Malthusian work at the beginning of the 19th century was widely reviewed and discussed, specifically among Genevan naturalists, such as the works of Augustin de Candolle and the Delesserts (Macdonald 2017, p. 14).^7^ In particular, the elder de Candolle’s interpretation of the balance in nature presented by Malthus in the field of economics, and its application to a biological model such as plants, was the notion that later influenced the thinking of Charles Lyell, Charles Darwin (Bowler 1976, p. 632; Corsi 2004, p. 233), and without a doubt, in his son Alphonse.

Another aspect that draws attention in the 1836 publication is that Alphonse de Candolle gave greater relevance to the environment in determining geographical distribution limits, compared to his two later works. What is surprising is that he overlooked that his father Augustin de Candolle (1820), in his essay *Géographie botanique*, had already overcome this environmental determinism–so widespread and so in line with natural theology – by explaining that the spatial distribution of organized beings was not exhausted by current circumstances. Other historical causes, probably geological, that no longer exist today could explain the *habitations* (Nelson 1978, p. 281).

Alphonse de Candolle claimed that two sets of causes relate to the establishment of cultivated species: (1) physical causes (which are the same for wild species) such as temperature, light, humidity, etc., and (2) human causes (which are variable according to the species cultivated) such as rituals, industry, trade, etc. These two components determine the spatial distribution of cultivated plants. Although both causes were interrelated in the history of domesticated plants, the physical causes were addressed by the botanical geography approach. Thus, even in cultivated plants, it was possible to estimate the approximate limits, especially if cultivation occurred in the origin regions. Since physical factors acted equally on wild and domesticated plants, it seemed logical to assume that the domestication of some plants must have started in their original area of distribution since, in this area, the species in question would be more abundant.

In considering these characteristics, Alphonse de Candolle pointed out the undeniable relationship between the laws of botanical geography and management systems in plant domestication. Proof of this is the correspondence between specific taxa, the physical conditions in their distribution areas, and the ease or difficulty of their cultivation. He also indicated the existence of taxonomic groups with few or no species that have been cultivated and explained this based on the traits of these botanical families and their relationship with living conditions and with the area of wild distribution. For example, the families Melastomaceae, Myrtaceae or Rubiaceae, etc., have typical species restricted to scorching and humid environments, which makes their cultivation difficult. In the words of de Candolle, “[b]otanists admit that the most endemic species are the most difficult to cultivate” (de Candolle 1836, p. 12).

In order to demonstrate that the factors that determine the distribution of wild species also determine the distribution patterns of domesticated species, Alphonse de Candolle considered that locating the sites of origin of cultivated plants would account for a common origin between the cultivated and wild populations of the present day, at least on the assumption that the area of origin and the beginning of their domestication are in the same area. However, although this assumption was implicit in his arguments, he neither spelled it out nor supported it on further botanical or geographical evidence.

In *Distribution géographique des plantes alimentaires* (1836), de Candolle made observations about the differences between wild and cultivated plants, which today seem vague and limited to mere descriptions, since he did not propose general principles regarding these differences and the processes underlying the differentiation between wild and domesticated. He did not go beyond pointing out that the effects of the environment on variation and geographical distribution are the same in plants under natural and cultivated conditions. Although this generalization that de Candolle proposed could represent a modern interpretation of the domestication process since it implies a shared geographical and genealogical origin among the existing specimens (wild and domesticated) of a species, the author’s thinking only referred to a history of cultivated plants without committing to an expanded explanation of all species and their origin before domestication.

Almost 20 years after his *Distribution géographique des plantes alimentaires*, de Candolle published *Géographie botanique raisonnée* (1855). Of course, during that intermediate period, he continued to publish texts on the taxonomy of different botanical families, reports on the geology and vegetation of different regions, and contributions to the multivolume work started by his father, *Prodromus systematis naturalis regni vegetabilis* (1824–1873).

During the 1850s, the evolutionism debate had already posed different theoretical challenges. Several exponents across Europe had published works focusing on the transformation of species as a natural process to explain issues such as adaptation, biological diversity, and extinction (Corsi 2005; Galera 2016; Friedman and Endress 2020). All this subject matter was familiar to Alphonse de Candolle, and he understood that the central question had to do with how the modifications of organized beings had come about, through a material connection of successive beings, or the creation of new forms independent of the previous ones (de Candolle 1855, p. XIII). Explaining and demonstrating the mechanism by which transformations occur was indispensable to legitimize any theory. Lamarckian theory was the most successful and widespread transformist proposal during the first half of the 19th century, although it was not the only one. There were other emerging proposals represented a direct complement to what Lamarck proposed (Galera 2016, p. 16).

For Alphonse de Candolle, Lamark’s theory was an exaggerated view that failed to contradict the fixity of species convincingly (de Candolle 1855, p. 1075). However, he did not categorically deny the possibility of transformation, although he thought it could occur only exceptionally, but not as a general process. In the preface to the *Géographie*, he began by stressing that the present geographical distribution of plants was the result of climatic and geographical changes over time and emphasized the need not only to describe but also to investigate how much of the present distributions could be explained by recent causes and how much by earlier causes (de Candolle 1855, p. XII). This reaffirms that for de Candolle, the historical dimension was essential to understanding the natural world. In turn, his interest in delving into past causes implicitly involved the question of the very origin of plant species. However, de Candolle did not fully address this issue (de Candolle 1855, p. XV).

In his 1855 book, he dedicated a chapter to domesticated plants, entitled “Origine géographique des espèces cultivées,” where he expanded the number of cultivated species to 157 (de Candolle 1855, p. 984). A significant difference from his earlier work of 1836 was the way he approached classification. Instead of considering a natural system *sensu* Jussieu, he did so by taking into account the useful traits of each cultivated plant and paying special attention to the degree of modification they had undergone, intending to trace their wild origin. Alphonse de Candolle reasoned that the possibility of reconstructing the history of a cultivated species implied finding out the uses of the parts of the plant, how its cultivation was managed, and fundamentally, it was essential to know the variability of each form. The variation present in the structures of individuals and the variation within a species were criteria that Darwin later used in his analysis of domestication (Darwin 1859); in fact, he came to assume that domesticated productions were even more variable than the organisms that remained in the wild conditions (Darwin 1868, p. 253).

Variation within cultivated species was a crucial element in de Candolle’s work. The more varieties of a plant were known in an area, the more likely it was to be its place of origin (Vavilov 1992, p. 28; Cubero et al. 2009, p. 28). Alphonse de Candolle’s theoretical and methodological proposal for approaching the geographical origin of domesticated species consisted of comparing the distribution of species of the same genus. This would indicate a hypothesis of historical relationship in the occupation of distribution areas. The closer the taxonomic relationship of a group of species, the closer their geographical distribution was likely to be. However, this criterion could not be applied in cases of transportation and establishment of plants in areas far from their original distribution for marketing reasons, a practice that has intensified significantly since the end of the 18th century (Anderson 1992, p. 140). Therefore, the most significant number of varieties could indicate the place of origin only if the native forms of an entire region were considered. This supported what the Darwinian paradigm later proposed: “The number of varieties or breeds is still an indicator. The more different modifications a cultivated species offers in one part of the globe, the more likely it is that it is ancient and native there” (de Candolle 1855, p. 980).

Another topic he discussed in his *Géographie* (1855) was the difficulty of distinguishing between species and varieties. Towards the 1850s, debates on the delimitation of species (consequently varieties) produced a theoretical and methodological lack of consensus among taxonomists that resulted in a division of approaches between those who defended a broad species concept (*lumpers*) and those who preferred narrowly delimited species (*splitters*) (Bonneuil 2002, p. 7).

During cultivation, a wide range of forms became evident, depending on the approach to delimitation, and some of them were identified as different species. A notion of species that considered the varieties within the same category (lumper approach) was more practical and contributed to the solution to the epistemic crisis within the taxonomy. de Candolle seems to have adopted this broad approach to the species since the variability observed in wild and domesticated plants was an always present attribute, so naming “new” species only considering morphological variation represented an imprecise practice.^8^

One of de Candolle’s contributions to this debate can be seen in the distinction between the concepts of *race* and *variety*. Races can transmit their traits to their offspring, unlike varieties, whose distinctive traits are lost in their offspring or even have their descendants revert to their original form. He wrote:

One thing struck me tremendously in the methodical research I have just carried out. It is the antiquity of several races or hereditary varieties of our cultivated plants. Cultivation is probably thought to have too great an effect. Because it can create forms transmissible by seeds, it is believed that this phenomenon has happened often and that the main changes are due to this cause. The fact is that cultivation produces many minor modifications [i.e. varieties], while truly hereditary and clear-cut races are almost always ancient; that is, they date back to a time of which man has lost track. (de Candolle 1855, p. 989)

It is worth noting that this distinction implicitly suggests the idea that the traits that distinguish varieties do not manage to transcend the species limit since they are inconstant or depend on human intervention to replicate over time: “Varieties can become races. They all have a certain tendency to become so; but they encounter in nature a host of obstacles which make the constitution of races independent of extremely rare” (de Candolle 1855, p. 1084).

De Candolle used the concepts of *race* and *variety* to make a distinction in the context of domestication. The main difference was the constancy of form through the inheritance of characters. The races have managed to establish these traits as necessary for their cultivation; the varieties, on the contrary, do not present this uniformity in the useful characteristics from generation to generation. For this reason, he concluded that races are older than varieties. He also pointed out a difference between the variation that occurs naturally in wild plants and the variation that arises in domesticated forms since the former is more consistent, while that produced by cultivation is more labile.

The term *race* and the concept of *species* that de Candolle developed in *Géographie* are very similar since an important component is the inheritance of traits specific to each taxon. However, his definition of species was more complex since it applied interchangeably to domestic and wild forms and referred to aspects such as interfertility, phenotypic constancy (through inheritance), and the natural variation that arises between individuals that come from the same lineage. He wrote:

The species is a collection of all individuals which are sufficiently similar to offer the following conditions: 1° almost always fertilize each other with ease and give ordinarily fertile products (when it comes to Phanerogams); 2° preserve their common, current characteristics from generation to generation under varied external circumstances; 3° present from one individual to other only differences in form and physiological nature similar to those which are observed by comparing several individuals which we know positively to have come from common stock in the species, or even, if you wish, in species close enough to each other so that a comparison is not forced. (de Candolle 1855, p. 1071)

Alphonse de Candolle considered his species to be real entities, made up of groupings of similar individuals, capable of generating fertile descendants similar to their parents but tending to vary due to their relationship with the local environment. Alphonse de Candolle highlighted the observable variability of species and considered it a necessary attribute to define them (Wilkins 2003, p. 16). Variation was relevant to his species conception, but this did not necessarily represent a transformist approach. However, de Candolle stated that he intended to outline a sufficiently general definition that could be adapted to any theory because science can validate explanations that previously seemed improbable (de Candolle 1855, p. 1071).

 He did not believe that spontaneous variations could be maintained and perpetuated to form new species, but that they are generally lost or reverted to their aboriginal stock. It is therefore not surprising that Darwin, who was well aware of de Candolle’s views on transformist ideas, commented to him in his first letter of November 1859: “I am perfectly well aware that you will entirely disagree with the conclusion, at which I have arrived.”^9^

Although de Candolle, like Darwin, drew attention to the remarkable and discontinuous intraspecific variation typical of cultivated plants, taxonomists of the time frequently included these varieties within the category of species (Pickersgill 1977, p. 591; Bonneuil 2002, p. 3), generally based on criteria such as morphological similarity and interfertility (Wilkins 2009, p. 122). Unlike Darwin, de Candolle argued that species were stable entities, so new species were unlikely to form. However, he admitted that over long periods and as long as they remained isolated, races could be modified to form “quasi-species” (de Candolle 1855, p. 1096). Without isolation, the new distinctive features disappeared when crossbreeding with other individuals.

De Candolle had understood that despite the observable differences between a wild and a domesticated type, the morphological criterion was not always decisive in establishing a specific identity. The difficulty in establishing the particular identity of cultivated plants was also related to the distribution area in which each species originates. The farther the ranges are from the hard-to-identify forms, the more likely they are to be different species. However, this criterion may have exceptions and may be subject to the domestication history of the species in question.

The history of domestication is different for each species, although it is likely that similar cropping systems have been followed for several plants. In a management context, some contingencies are not controlled, such as the presence of other plant species whose development is favored in the growing conditions (more fertilizer, availability of water, protection from herbivores, etc.). Generally speaking, the closest wild relatives of a cultivated plant are usually weeds of the same or a closely related species. The distinction between the actual wild ancestors and the wild descendants of a cultivated plant was a relevant issue in de Candolle’s interpretation of domestication as a historical process (Pickersgill 1977, p. 592). These plants were unintentionally introduced, growing alongside the plants of interest and were initially undesirable in cropping systems (as weeds). Later, it was realized that they could be integrated into domestication if humans obtained some benefit from them. This indirect way in which some plant management begins could be another model of how domestication originated. With the vast evidence he reviewed, de Candolle concluded that most modern domesticated plants had originated intentionally. However, it is considered that even under controlled conditions, there can be species that appear spontaneously in fields, but represent a benefit and then eventually become cultivated consciously. This was important in his argument to explain that due to the reproductive isolation (by spatial isolation) between cultivated and wild plants, as well as the preference of valuable variations to some human purpose, after numerous generations results in a differentiation of form. He wrote that “[i]t is not the cultivation itself that changes them, but rather the care with which the seeds of the plants are isolated in the cultivation, offering certain helpful or curious modifications to sow them apart and to repeat this choice successively for several generations” (de Candolle 1855, p. 992).

It is striking that in this quotation from a work published in 1855, de Candolle seems to outline a general scenario in which selection and isolation would play an essential role so that the modifications produced by domestication could be preserved over time, a scenario that would also appear later in *Origin of Species* (Darwin 1859). This suggests that the study of domestication had an essential role in generating hypotheses and theories about biological evolution, even independently and before Darwin’s work.

The conclusions de Candolle reached in his *Géographie botanique raisoneé* on the subject of the origin of species are listed here and can be considered conservative. First, species are susceptible to modifications due to unknown internal causes and weak external causes. Second, the new forms produced become heritable only due to the effect of isolation and time. Therefore, new species would have to habitate separate areas. Third, the formation of new species by derivation would be extremely rare; the vast majority arise independently (de Candolle 1855, p. 1098).

In the conclusion of his *Géographie*, de Candolle reiterated the importance of this research program as a strictly scientific approach to the origin of cultivated plants, based on evidence and examined from the evidence of botanical geography. He wrote:

When we study all the species one by one, without neglecting those which are less attractive than wheat or potatoes, but taking into account all the crops, we see that, year by year, we have arrived at safer and simpler notions about the origin of the species and their spontaneous state. Instead of supposing immense modifications in specific forms, or disappearances of continents, or miraculous acts, we arrive at the very ordinary idea that the progress of geographical and botanical discoveries will probably make it possible to gradually note the original homeland and the primitive state of all cultivated plants. (de Candolle 1855, p. 984)

In this conclusion it can be seen how, despite a moderate acceptance of modification in domesticated forms, de Candolle seems to distance himself from explanations that affirm the emergence of new species from previous forms–at least without sufficient evidence and without the theoretical structure that could sustain such hypotheses. It seems ironic that de Candolle’s treatise anticipated the perspective of *Origin of Species* (1859), just before Darwin published his great work (Egerton 2019). It is evident that de Candolle already had a marked interest in the subject of the origin of species, and his position at this point seemed to lean towards transformism. However, he preferred to be cautious, partly because he felt that more solid evidence was lacking and partly because he feared the discredit that a transformist position might cause him within orthodox intellectual spheres. In this indecision, he made somewhat ambiguous arguments that increased the tension in his ideas about the species.

### Correspondence with Charles Darwin and the Acceptance of Natural Selection

As far as the historical record allows us to know, Charles Darwin and Alphonse de Candolle met in person in late May 1839, thanks to a common connection with John Henslow, although there are no further details on that meeting (Baehni 1955, p. 110; Macdonald 2017, p. 21). The epistolary relationship between the naturalists began in November 1859, when Darwin wrote to de Candolle to notify him that he was sending a copy of *Origin of Species,* since Darwin frequently resorted to the *Géographie botanique* in his discussion of geographical distribution.^10^ Darwin relied on the thorough and extensive research of de Candolle to support the thesis of his Chapters XI and XII, namely, that however peculiar the geographical distribution of individuals of the same or closely related species might be, it could be explained by migration from the same ancestral source and subsequent modification (Darwin 1859, pp. 407–408). De Candolle’s reply came as late as June 1862, in a missive setting out his position on natural selection. He made it clear that his view was similar to that of Asa Gray, i.e., although he would like to believe in his theory of descent, convincing empirical evidence was required to support the existence of natural selection: “The general hypothesis of an indefinite transmission of forms through the centuries, with more or less serious modifications, seems preferable to any other, but whether natural selection is the mode, that is what is vague in my mind.”^11^ Four days later, on 17 June of the same year, Darwin replied to de Candolle’s letter and took the opportunity to reiterate his admiration for the *Géographie botanique*. He also reiterated that he was not surprised by his reticence towards natural selection:

I am not at all surprised that you are not willing to admit natural selection: the subject hardly admits of direct proof or evidence. It will be believed in only by those who think that it connects & partly explains several large classes of facts: in the same way opticians admit the undulatory theory of light, though no one can prove the existence of ether or its undulations.^12^

In the same year, de Candolle published *Étude sur l’espèce à l’occasion d’une révision de la famille des cupulifères* (1862), a paper that synthesized the information on holm oaks and oaks to be included in the *Prodromus*. In this new publication, de Candolle allowed himself to describe the Darwinian theory as “[t]he most modern, and at the same time the most ingenious and the most complete of systems based on an evolution of beings organized within a series of times” (de Candolle 1862, p. 57).

He was more willing to accept Darwin’s theory, albeit more as a brilliant hypothesis than as fact. He again expressed severe reservations about natural selection. He maintained his previous argument about the transformation of species, i.e., it was not the emergence of new forms that was difficult to accept, but the mechanism that allowed their preservation over time in a particular environment. Although the theory of natural selection could explain many facts, de Candolle thought the evidence in its favor was insufficient, often questionable, and had not been proven by direct evidence.

In particular, de Candolle considered that Darwin had not taken into account an important aspect, the so-called “loi de balancement,” or the law of organ balance, which had already been widely developed and discussed by various naturalists (Appel 1987, p. 151; Nyhart and Lidgard 2011, p. 381).^13^ De Candolle expressed this concern, as follows:

Mr. Darwin agrees, moreover, that modifications useful to the species are rare; that many others are useless and therefore transitory. We might add that by a known law of balancing organs and functions when a useful modification exists on one point of the being, it results in a modification in the opposite direction on another point. (de Candolle 1862, pp. 62–63)

Thus, even if an advantageous variation arose, it would not generate the appearance of new species because of deleterious modifications elsewhere. Unlike Darwin, de Candolle refrained from extrapolating the changes occurring in domesticated species to wild species.

A decade later, in a letter he sent to Darwin on January 1873,^14^ de Candolle still maintained that the law of balancing prevented the preservation and evolution of advantageous variants. According to him, the different phenomena that can produce variation tend to be neutralized, causing modifications to be extremely slow or negligible so that it would be practically impossible for new species to be generated from new variants.

In the same year, in his book *Histoire de la science et des savants depuis deux siècles*, de Candolle openly described natural selection as a fact, not just a theory: “Selection is neither a theory nor a hypothesis, it is the expression of a necessary fact” (de Candolle 1873, p. 10). However, he still doubted that this is a widely prevailing process, as it requires two conditions to occur: first, “[t]hat a new form has escaped external influences for a few years, from atavism and fertilization by old forms to the point of having become hereditarily distinct,” and second “[t]hat it offers relative advantages, which make it better adapted to surrounding circumstances, without however being more poorly organized in other essential respects, such as reproduction, for example” (de Candolle 1873, p. 10).

Almost ten years later, de Candolle published the work entitled *Darwin* c*onsidéré au point de vue des causes de son succes et de l’importance de ses travaux* (1882). This text, published in the year of Darwin’s death, shows a remarkable change in de Candolle. It is essentially a celebration and recognition of the English genius, describing him as noble, modest and kind. What is interesting is that de Candolle acknowledged that as early as 1855 he had admitted, at least in some instances, the creation of new forms derived from old ones: “The current distribution of plant species, considered particularly in the islands, forced me to admit, in 1855, four years before Darwin’s first theoretical work, a creation, in certain cases, of new specific forms derived from old ones” (de Candolle 1882, p. 14).

The joint consideration of taxonomic, palaeontological, organogenetic facts, as well as Lyell’s concept of deep time, no longer allowed him to accept the view of species as almost immutable forms. According to de Candolle, Darwin’s genius was to integrate all this knowledge under the theory of transformism explained by its cause, natural selection. With this reinterpretation, de Candolle ended up recognizing selection as an essential factor in explaining evolution.

Twenty-eight years after the *Géographie* (1855), de Candolle expanded his research on cultivated plants, their distribution, and their origin in a significant book, *Origine des plantes cultivées* (1883). This work reviewed the taxonomic status and geographical, historical, and linguistic information of 247 cultivated species. He traced 199 Old World species and 45 New World species back to wild ancestral forms. Three additional species were considered of uncertain origin (de Candolle 1883, p. 362).

In the second half of the 19th century, with the rise of the evolutionary debate, new theoretical interpretations began to emerge about the biological notion of the species (Wilkins 2009, p. X; Richards 2010, p. 97). Before Darwin’s theory of descent with modification, determining the origin of cultivated species consisted more of taxonomic categorization through morphological comparison than historical reconstruction of the dispersion and naturalization of domesticated species. Thanks to the notion of common ancestry incorporated in Darwinian thought, the concept of species became more complex by acquiring a historical dimension, and this was reflected in the interpretations of authors such as Alphonse de Candolle.

In this last, late work on cultivated plants, de Candolle showed a greater propensity towards evolutionary thought, specifically the Darwinian proposal. Initially, de Candolle’s conception of the origin of domesticated species allowed him to consider scenarios in which modification of organisms occurs under special circumstances, but he never explicitly committed to a theory. However, starting in 1859 with the publication of *Origin of Species*, Darwin’s explanation took a leading place in de Candolle’s recurring discussions on the evolution of organisms. He carried out a prolonged scrutiny for more than 20 years as reflected in the different texts we have mentioned. Through a sustained correspondence with Darwin, the Swiss botanist became convinced of the transformation of species by natural selection.

It is important to say that de Candolle’s arguments do not reveal why he leaned towards the transformist approach. However, his approach to interpreting several issues, such as the degree of variation and its relationship with geographical distribution, reproductive isolation caused by physical isolation, and the temporal parameter, were gradually modified by de Candolle, as he integrated evolutionism into his studies of cultivated plants.

From the beginning of *Origine des plantes cultivées* (1883), de Candolle acknowledged that knowledge about the original distribution of cultivated plants was erroneous or incomplete. Another aspect that remained unknown was how long species had been domesticated. In this work, its objectives focused on determining the geographical origin of the cultivated forms, as well as estimating the approximate time that such species had been cultivated. Of the species whose wild ancestor was known, the minimum time was 2000 years and the maximum 4000 years or even longer. In the cases of cultivated plants whose ancestors were unknown, de Candolle proposed two explanations: “(1) either these plants have changed their form in nature, as in cultivation, since historical times, in such a way that they are no longer recognized as belonging to the same species; (2) or they come from an extinct species (de Candolle 1883, p. IV).

Thanks to the development of evolutionary thinking since *Origin of Species* (1859), it was possible to reframe the idea of domestication as an evolutionary process. In 1883, de Candolle was doing so by integrating new questions into his research on cultivated plants. Among these further questions, he asked how and when the initial cultivation events occurred for different species, thinking of degree of domestication as a spectrum. In particular, to answer how domestication occurred he resorted to the Darwinian concept of selection: “[*s*]*election*—that significant factor which Darwin had the merit of introducing so happily into science—plays a vital role once agriculture is established, but in any age, and especially in the beginning, the choice of species is more important than the selection of varieties” (de Candolle 1883, p. 2).

After this development in his interpretation of domestication and evolution, de Candolle could articulate the question of geographical space in the historical explanation of the origin of cultivated plants. Like wild species, the species that have been cultivated have a geographical center of origin, so it seemed to de Candolle that the most plausible hypothesis was that the domestication of the species in question also began in the original space:

The various causes that favor or hinder the beginnings of agriculture explain why some regions have been populated for thousands of years by farmers, while wandering tribes still inhabit others. Rice and several legumes in South Asia, barley and wheat in Mesopotamia and Egypt, several Paniceae in Africa, and corn, potato, batata, and cassava in America were promptly and easily cultivated, thanks to their obvious qualities and favorable climatic circumstances. Centers were thus formed from which the most valuable species spread. (de Candolle 1883, p. 3)

De Candolle used both extensionist and dispersionist hypotheses, *sensu* Fichman (1977), to explain phytogeographical patterns. Extensionism accepted the existence of ancient land areas now submerged, while dispersionism asserted that the configuration of continents and oceans had remained largely unchanged, at least since the Tertiary. Darwin, for his part, was a fierce opponent of extensionist hypotheses (Fichman 1977, p. 45). In a letter that Darwin sent to de Candolle in January 1863, he told him: “I really think that I believe in as much migration as even you believe in, & as shown in your admirable great work; only I do not believe nearly so much in continental extensions & I believe more (not very much more, I begin to suspect, & it pleases me greatly) than you do in modification in form.”^15^

In *Origine des plantes cultivées* (1883), de Candolle continued to accept both explanations. He leaned towards large terrestrial extensions to explain the distribution of wild plants, while for cultivated plants, he preferred dispersal, supported by abundant evidence of organisms that had been domesticated and had had humans as their primary dispersers, both purposely and unconsciously:

Each species usually has a continuous or vaguely defined home. However, sometimes, it is disjointed; that is to say, the individuals who compose it are divided across remote regions. These cases, which are very interesting for the history of the plant kingdom and the terrestrial surfaces of the globe, are far from forming the majority. Therefore, when a cultivated species occurs in the wild, very abundantly in Europe and less abundantly in the United States, it is likely that, despite its appearance being native to America, it has become naturalized there because of some accidental transport. (de Candolle 1883, p. 9)

The hypothesis that cultivated plants were domesticated in centers of species diversity was initially proposed by Alphonse de Candolle (later extended by Nikolai I. Vavilov) (Smýkal et al. 2018, p. 3). De Candolle proposed three different centers of origin of agriculture, taking the area where the cultivation of indigenous plants had begun as a criterion. These regions, identified as the great centers of domestication, coincided with the location of the great centers of civilization. Of course, he pointed out that the domestication of plants had emerged in other territories also. However, in those minor centers it did not coincide with establishing economies and cultural centers based on domesticated species. De Candolle concluded that the cultivation of most domesticated plants began within their original range. He wrote:

Agriculture emerged in ancient times, at least as far as the main species are concerned, from three large regions where certain plants grew and which had no communication with each other. These are China, southwest Asia (linked with Egypt), and intertropical America. I do not mean that in Europe, Africa, or elsewhere, wild peoples did not cultivate some species, at a remote time, in a local way, as accessories for hunting or fishing. Still, the great civilizations, based on agriculture, began in the three regions that I have just indicated. (de Candolle 1883, p. 13)

The reinterpretation of domestication as an evolutionary process raised a question about the specific identity of organisms under domestication since there is a variation directly related to their management, and even the divergence of traits between domesticated and their wild counterparts was accepted. The answer to that question had been discussed since the 18th century with taxonomic classification systems, as in the case of Linnaeus, who described and classified domesticated animals and plants as independent species and consequently gave them distinct names. In some cases, a domesticated form was the species that typified an entire genus (including wild species). This debate took a different direction with the development of evolutionism (Ritvo 1992, p. 365; Zeller and Göttert 2019, p. 3).

As noted, in 1883 de Candolle accepted that cultivated plants had been modified to some extent. Still considering the evidence, his final answer was that they belong to the same species of the wild type:

I believed, a priori, that a much larger number of species cultivated for more than 4000 years would have deviated from their ancient state to such a degree that we could no longer recognise them among spontaneous plants. On the contrary, it appears that forms prior to cultivation were usually preserved alongside those that farmers obtained and propagated from century to century. Two causes can explain this: 1° The period of 4000 years is *short relative* to the duration of most specific forms in phanerogamous plants. 2° Cultivated species receive, outside of crops, incessant reinforcements through seeds that man, birds, and various natural agents disperse or transport in a thousand ways. The naturalizations thus produced often make it easy to mistake the wild plants for those from cultivated plants, especially since they fertilize each other since they are of the same species. (de Candolle 1883, p. 369)

Several important aspects of de Candolle´s assimilation of evolutionary thought stand out in this last reference. As can be seen, he claimed that cultivated plants, although modified by domestication, still belonged to the same wild species. This can be interpreted as a conservative position since, in reality, it does not dare to affirm that cultivation has modified the organisms so much that it allowed the emergence of new species. However, the question of time may allow us to make another reading of de Candolle’s statements. He suggested that 4000 years would be an insufficient period to generate divergence between species under domestication. Still, by considering that for wild plants, the time scale was much older than any human record, he left open the possibility of historical processes that contribute to the modification and speciation of lineages (Mïkulinsky 1974, p. 227). The basis for estimating the idea of deep time was geological evidence and its integration into the evolutionary discussions of the 19th century (Bomio and Robert 1887, p. 342; Browne 1992, p. 471). So, in the vision proposed by de Candolle, like that of Darwin, the geological scale was a component that would allow us to understand the antiquity of the species and the scenarios in which they would have developed, either modifying to the point of originating new species or becoming extinct.

It is undeniable that regarding cultivated plants, de Candolle moderated his interpretation of transformation and preserved a broad notion of the species, including the different domesticated races as part of the same taxon. This decision makes sense when considering the criteria with which he had defined the species since 1855 (interfetility, phenotypic similarity by inheritance, and variability). Cultivated forms could meet these parameters and, therefore, be considered members of the same species as wild organisms. However, the discussion on the time of domestication and the origin, not only of the cultivated form but of the wild ancestor, shows that de Candolle was leaning his conclusions towards an evolutionary framework, specifically Darwinist, since he did not seriously discuss any other proposal of organic change, even though he knew the proposals published by Jean-Baptiste Lamarck, Alexander Moritzi (Friedman and Endress 2020), and Dominique A. Godron (Galera 2016).

Having considered this review, we can understand that de Candolle did not convert to Darwinism immediately after reading *On the Origin of Species*. The change was not a smooth process but rather one of reluctance and a persistent rejection of natural selection. Before 1882, strict adherence to empirical data prevented de Candolle from accepting the generation of new species from the transformation of old forms. He considered any theory of evolution in organic beings to be fundamentally speculative, as there was no body of hard evidence to prove the veracity of such explanations, including Darwin’s. However, de Candolle’s thinking coincided with important aspects of Darwinian theory: the idea of organism-environment fit through historical processes; the idea of common ancestry; the importance of variation and its relation to the geographical distribution of species;’ the struggle, or war, between organisms for all kinds of resources; selection as a molding process; the notion of remote time; and even the interest in discovering a mechanism capable of explaining a constant inheritance of characteristic traits in races and species.

One of de Candolle’s most frequent criticisms of Darwin’s proposal was using domestic types as models for wild forms. According to de Candolle, it was the other way around. What occurred in wild species was perfectly applicable to domesticated organisms. What is certain is that both authors shared a historical view of domestication that implied changes. The conclusions of each were different. Darwin, for his part, was adamant about interpreting variation in domesticated species as the beginning of a process that eventually led to the formation of new species. De Candolle, on the other hand, underwent a three-stage transition during his long study of plant domestication: anti-transformism, moderate transformism, and finally, full Darwinism. Through the intense debates that Darwinian theory provoked in the last decades of the 19th century and the intense correspondence he maintained with the English scholar, de Candolle was convinced of the great power that natural selection had to explain the domestication process.

At first glance, the intellectual positions between an anti-transformist de Candolle and a transformist Darwin seem opposed. However, a closer look changes that perspective. Therefore, it is crucial to examine what aspects and at what times they coincide. While it is true that there is a similarity of ideas between Darwin (1868) and de Candolle (1883), one may note that de Candolle’s reflections on the transformation of living beings had emerged from an earlier moment, in *Distribution géographique des plantes alimentaires* (1836). In that work, de Candolle began to sketch the idea of the struggle for resources between species and the question of the abundance of individuals as a critical aspect of that competition. Likewise, ancestry and geographical origin were present from the beginning of his work on the subject, although there was no conceptual unification of all these components in explaining the origin and history of species.

In *Géographie botanique raisonné* (1855) de Candolle again described the struggle for existence and competition between species. Those whose distribution is wider tend to expand even more and end up displacing other species, increasing the chances of extinction for those harmed by competition (Mikulinsky 1974). This selectionist notion is also carried by de Candolle in his investigation of cultivated species since he considered that the selection of useful or unusual variations and their consequent reproduction (separated from individuals lacking such a trait) is the necessary process to domesticate some species plant or to create new varieties. Darwin agreed with these ideas: “[Alphonse] de Candolle and others have shown that plants which have extensive ranges generally present varieties; and this might have been expected, as they become exposed to diverse physical conditions, and as they come into competition” (Darwin 1859, p. 53).

De Candolle’s work of 1855 may have represented a key antecedent for understanding the reception and development of Darwin’s theory in the decades following the 1859 publication of the *Origin of Species*. Likewise, in 1855, Darwin began experiments and observations with pigeon breeding, and had maintained communication with various crop and horticultural experts since at least 1839 (Evans 1984, p. 125; Feeley-Harnick 2020, p. 150). It is interesting to note how the study of domesticated species independently attracted the attention of contemporary naturalists, whose conclusions about the mutability of species came to be similar.

Another aspect that stands out in the *Géographie botanique* (1855) is the notion of species, which de Candolle began to complexify. In his previous work, *Distribution géographique des plantes alimentaires* (1836), he constantly refers to species as real entities that manifest responses to environmental variations. The most general idea of species that this author developed was from the similarity of individuals to their parents. He did not delve into any criterion that would allow the practical identification of species until 1855.

The species concept that de Candolle seems to support from the *Géographie botanique* refers to a group of individuals that, due to their resemblance to each other, must descend from a shared ancestral type (Wilkins 2009, p. 127). This more specific interpretation assumes that the origin of cultivated plants is in some wild ancestor, representing a genealogical criterion for identifying species. On the other hand, it also suggests that the distribution area of that ancestor contains the original distribution area of such a cultivated form, which implies a geographical identification criterion.

Throughout his productive career in botany, Alphonse de Candolle always kept the role of domesticated plants in mind. From an early approach in 1836, the variations observed between different races of the cultivated species and between some wild forms allowed this naturalist to begin a profound reflection on the relationship of kinship between these varieties, even without the Darwinian proposal.

In his obituary dedicated to Darwin (de Candolle 1882) and in his work devoted exclusively to cultivated plants, *Origine des plantes cultivées* (1883), de Candolle exhibited a complete adherence to Darwinian thought by integrating aspects such as the time of domestication, how plant species have been domesticated, and the coincidence between the centers of domestication with the original distribution areas of the wild types. However, his change was more declarative than argumentative. He simply no longer insisted on his arguments contrary to Darwin’s proposal. In the end, the explanatory power of the theory of descent with modification convinced him. Undoubtedly, one more reason for this change in de Candolle’s interpretation was because the debate generated by the theory of natural selection invited reflection on the history of species and their relationship with geological history.

In Alphonse de Candolle’s thought, the current distribution of species implied a historical conception of living beings and the environments they have occupied (Caponi 2008, p. 38). Therefore, the historical reconstruction of the distribution area for each cultivated plant necessarily led de Candolle, over almost five decades, to reflect on a model that explained the natural variation of organisms, the relationship with physical conditions (and the change of these conditions over time), the reproductive isolation that occurs between organisms in captivity and wild specimens, and the phenotypic modifications that are generated.

In the 1883 work *Origine des plantes cultivées*, de Candolle presented a completely transformist position. From his analysis there, he was able to reach some main conclusions. First, the traits that distinguish the cultivated type from the wild type have been generated by the selection of variations and their isolated reproduction. Second, the cultivated species belong to some wild species with which they share a common ancestor and geographical origin. Third, the center of geographical origin is usually the area where the domestication of such a plant began. Fourth, the time in which the modifications under cultivation occurred has been extensive (around 4000 years), and there is evidence to support the view that the same species in wild conditions existed long before this estimate.

During the decades leading up to that 1883 publication, de Candolle’s correspondence with Darwin was constant, so in addition to accepting the transformation of species, he at length accepted Darwin’s theory of natural selection. Both authors had done fruitful work that included numerous pieces of evidence that could support the explanations derived from their observations. Darwin’s thoroughness in considering significant elements and evidence is comparable to the scientific rigor of Alphonse de Candolle. These naturalists believed that observable variation in individuals was fundamental. Particularly in the case of de Candolle, data parametrization was paramount in his methodology and interpretation of biological phenomena, as demonstrated by the application of botanical arithmetic in his geographical studies (Browne 1980, p. 13).

### The Origin of Cultivated Plants after Darwin and de Candolle

Throughout the 19th century, particularly during the second half, there was a growing interest in domesticated species. In science, the questions that were investigated focused mainly on the wild origin of these species, their geographical origin, the heritability of the typical traits of a breed, and the process of modification from the wild to the completely domesticated. De Candolle’s work was part of this area of research, specifically on the geographical origin of cultivated plants.

Towards the end of this period, some authors pointed out the progress in investigating cultivated species that stemmed from the relationship between Darwinian theory and de Candolle’s methodological approach. For example, Édouard Dufresne (1883), in assessing the economic importance of a crop such as wheat, accepted the geographical origin proposed by de Candolle (Mesopotamia) and mentioned that the existence of multiple varieties of this widely cultivated cereal was undoubtedly evidence of the Darwinian mutability of the species.

Years later, Caullery (1897) published a review of the advances in the methodology of studying domesticated animals and plants, and considered that de Candolle’s *Origine des plantes cultivées* (1883) showed a theoretical continuity with Darwin’s *Variation of Animals and Plants Under Domestication* (1868); for Caullery both works focused on the search for the origins of domesticated species, on the genealogical reconstruction of these breeds, and on artificial selection as the process responsible for the distinction between wild and domesticated. Likewise, the Belgian botanist Théophile A. Durand wrote a biographical note a few days after the death of Alphonse de Candolle, in the *Bulletin de la Société Royale de Botanique de Belgique* (Durand and Errera 1893). It stated that de Candolle had been a Darwinist before Charles Darwin himself and this fact was known in England; it also stated that de Candolle’s 1855 publication discussed the possibility that current species were modified descendants of pre-existing forms. Durand also noted de Candolle’s attempt to apply Darwinian theories to humans, referring to the 1873 publication in which de Candolle sought to evaluate intelligence as a trait favored by natural selection in European scientists. However, in recent work, de Candolle’s contribution has been barely recognised, and sometimes he is not even mentioned (e.g. Brown 2010; Larson et al. 2014).

Judging from the marginalia in Darwin’s books, two authors greatly influenced him, Alexander von Humboldt and Alphonse de Candolle (Di Gregorio 1990, p. XXXIV). It is possible that de Candolle was more receptive to Darwin’s evolutionary proposal, starting in 1859, precisely because Darwin based his own biogeographical understanding of domesticated species on de Candolle’s work.

A separation can be seen in the theoretical interests of the transformists and those who focused on studying species and their relationship with the environment, such as Humboldt, Schouw, A.P. de Candolle, Alphonse de Candolle and others, who focused on the geographical distribution of plants and the environmental factors that govern this distribution (Acot 1983, p. 34). Although both positions involved the consideration of similar evidence, the study of the organism-environment dyad did not necessarily present a link with the transformist issue, even after the publication of *Origin of Species*. While Darwin tried to clarify the origin of species through a historical and anti-creationist approach, authors such as de Candolle sought to explain the limits of the habitations of species based on current parameters (e.g. temperature), migrations, disjunctions and their relationship with environmental changes due to geological changes. This led de Candolle to question whether these factors were permanent for each species or whether, on the contrary, species could change their physiological responses to climates different from that of their original area, over time. However, the fact that de Candolle resorted to geological and paleontological evidence, that is, historical evidence, probably facilitated his later acceptance of Darwinism (Acot 1983; Bomio and Robert 1887).

The question of centers of origin and the dispersalist model was significantly boosted by the publication of *Origin of Species* in 1859. In the later 19th century, with growing acceptance of Darwinian evolutionism, naturalists focused primarily on constructing models that explained the dispersion of lineages from their center of origin to their current distribution. The study of other primary spatial patterns (geographic gradients of biotic richness and replacement, geographic variation of phenotype, expansion-differentiation of unique lineages, and biogeographic homology) was replaced by reconstructing species’ centers of origin and dispersal routes (Juárez-Barrera et al. 2018, p. 1). Alphonse de Candolle’s publication, *Origine des plantes cultivées* (1883), clearly reflects this tendency in his thinking and research to clarify the centers of origin of cultivated plants within an evolutionary framework.

De Candolle´s notion of the center of origin of cultivated species and the identification of the center of maximum differentiation (in varieties) represents an antecedent of later biogeographic explanations, such as the concepts of center of diversity proposed by Nikolai Vavilov in 1926 or center of mass suggested by León Croizat in 1958, which means the area of greatest diversity of a particular taxon. Working at the Leningrad Institute of Plant Industry (Smith 1968), in 1926 Vavilov proposed eight primary areas of origin, which have come to be called centers of diversity or Vavilov centers (Vavilov 1992; Corinto 2014, p. 8; Hummer and Hancock 2015, p. 781). Vavilov’s regions included several areas proposed by de Candolle: Southwest Asia, China (East Asia for Vavilov), and Intertropical America (Central America and the Andes for Vavilov). Vavilov explicitly acknowledged the collaboration between de Candolle and Darwin as the most important precursor in the study of domestication (Vavilov 1992, p. 422).

Since then, the geographical study of cultivated plants integrated into an evolutionary approach has given way to the extended view of domesticated species as genetic resources (Müntzing 1959, p. 212; Chacón-Sánchez 2009, p. 355). Today, the intellectual heritage of the Swiss botanist has clear relevance due to the current context of climate change, food security, biodiversity loss, and the aid that an evolutionary approach can provide to resolve numerous urgent issues.

## Conclusion

Throughout his career as an expert in botanical geography, cultivated plants were one of Alphonse de Candolle’s recurring interests. Before learning about Darwin’s work, the Swiss botanist had already brought attention to the importance of knowing the center of origin of these domesticated species. After meeting Darwin and his work, and after having exchanged ideas for years, de Candolle proposed concrete hypotheses about the history of cultivated plants based on the available information. Abandoning the caution that characterized him previously, he likely decided to present such approaches because, by the end of the 19th century, the study of the domestication of species had extended beyond breeding and agriculture to the scientific arena. Likewise, the evidence considered legitimate was no longer limited to the descriptive botanical information of the species, but could include historical, archaeological, and linguistic data. Very likely, one more reason for this change in de Candolle’s interpretation was that the debate generated by the theory of natural selection invited reflection on the history of species and their relationship with geological history.

De Candolle, like Darwin, presented an analogy between wild and domesticated species by invoking essentially similar processes. And just as, for Darwin, the relationship between morphological variation and distribution was fundamental to support his theory of descent with modification, so too for de Candolle, the geographical distribution was a key aspect to elucidate the area of origin of cultivated plants. This contributed significantly to reinterpreting domestication as an evolutionary process in accordance with the Darwinian principles of variation, isolation, divergence, and modification. Thus, de Candolle integrated spatial distribution into the evolutionary interpretation of domestication.

As a factor in explaining the slowness of de Candolle’s full acceptance of Darwinism, it is possible that by the penultimate decade of the 19th century the transformist vision and Darwin’s theory permeated the scientific community, so that de Candolle no longer felt a great reputational risk by discussing transformist theory. It should also be noted that de Candolle was 76 years old when his obituary for Darwin (1882) was published. Perhaps in this work, he preferred to refine the subject that had fascinated him for so many years rather than re-enter the controversy of transformism. It is also possible that he did it as a tribute and a final recognition of the man he admired and respected. Whatever his motivation, de Candolle’s *Origine des plantes cultivées* (1883) was an original contribution that today retains fundamental importance for the life sciences. Before Darwin’s theory, determining the origin of cultivated species consisted more of taxonomic categorization through morphological comparison than historical reconstruction of the dispersion and naturalization of domesticated species. Thanks to the notion of common ancestry introduced by Darwinian thought, the concept of species became more complex, and this can be seen in interpretations of domestication such as that of Alphonse de Candolle.

## Footnotes

1 According to Gissis (2010), in early publications (before 1806) that contained some transformist notions, Lamarck attributed anatomical changes in organisms to a direct effect of the environment. From 1806, he changed this appreciation and considered that the impact of the environment was indirect, acting first on the needs of individuals, which in turn modified their habits, and consequently, the use and disuse of their structures.

2 Friedman and Endress (2020) point out that domesticated species in general were important in Moritzi’s discussion, particularly as evidence of morphological variation. In Moritzi there is no development between domesticated animals and plants and their geographical distribution.

3 All quotations from Augustin de Candolle and Alphonse de Candolle are translations made by the authors.

4 Although *Origine des plantes cultivées* is dated 1882, we use the second edition from 1883.

5 Darwin Correspondence Project: To Alphonse de Candolle, 11 November 1859, Darwin Correspondence Project, “Letter no. 2523,” accessed on 21 February 2024, https://www.darwinproject.ac.uk/letter/?docId=letters/DCP-LETT-2523.xml. Also published in The Correspondence of Charles Darwin, vol. 7. Alphonse de Candolle to Darwin, 13 June 1862, Darwin Correspondence Project, “Letter no. 3603,” accessed on 21 February 2024, https://www.darwinproject.ac.uk/letter/?docId=letters/DCP-LETT-3603.xml. Also published in The Correspondence of Charles Darwin, vol. 10. Darwin to Alphonse de Candolle, 17 June 1862, Darwin Correspondence Project, “Letter no. 3608,” accessed on 21 February 2024, https://www.darwinproject.ac.uk/letter/?docId=letters/DCP-LETT-3608.xml. Also published in The Correspondence of Charles Darwin, vol. 10.

6 The translation that Augustin de Candolle read was *Essai sur le principe de population* (1809), made by the Swiss philosopher Pierre Prévost.

7 Macdonald (2017) has emphasized the “British-French-Swiss networks” of the late 18th century and 19th century, as a means by which botanical material was distributed between institutions, which was fundamental to the development of the taxonomic and geographical knowledge. Likewise, debates circulated in these networks about the proposals of authors such as Rousseau, Malthus, the Delesserts, the Candolles, and Darwin.

8 Bonneuil (2002) has suggested that Alphonse de Candolle’s approach had tended to be more “splitty” until the early 1850s, taking into account the description of numerous species that were included in the *Prodromus.* However, the influence of Joseph D. Hooker, who defended a lumper position, occasionally influenced de Candolle’s interpretations towards the broad notion of species, for example in his treatment of the Cupuliferae (de Candolle 1862).

9 To Alphonse de Candolle, 11 November 1859, Darwin Correspondence Project, “Letter no. 2523,” accessed on 21 February 2024, https://www.darwinproject.ac.uk/letter/?docId=letters/DCP-LETT-2523.xml. Also published in The Correspondence of Charles Darwin, vol. 7.

10 Darwin to Hooker, 10 October 1855, Darwin Correspondence Project, “Letter no. 1763,” accessed on 21 February 2024, https://www.darwinproject.ac.uk/letter/?docId=letters/DCP-LETT-1763.xml. Also published in The Correspondence of Charles Darwin, vol. 5. (Darwin had previously expressed to Joseph Dalton Hooker his interest in the *Géographie botanique.*).

11 Alphonse de Candolle to Darwin, 13 June 1862, Darwin Correspondence Project, “Letter no. 3603,” accessed on 21 February 2024, https://www.darwinproject.ac.uk/letter/?docId=letters/DCP-LETT-3603.xml. Also published in The Correspondence of Charles Darwin, vol. 10.

12 Darwin to Alphonse de Candolle, 17 June 1862, Darwin Correspondence Project, “Letter no. 3608,” accessed on 21 February 2024, https://www.darwinproject.ac.uk/letter/?docId=letters/DCP-LETT-3608.xml. Also published in The Correspondence of Charles Darwin, vol. 10.

13 According to Appel (1987), this principle of organ balance had been developed in the 1820s by Étienne Geoffroy Saint-Hilaire and was part of a heated debate with Georges Cuvier throughout the 1830s. Specifically, Geoffroy resorted to the balance explanation to argue that the anatomical differences between species were the result of a balance between atrophy and the profit of the structures, which allowed their functionality to be maintained (or modified). Nyhart and Lidgard (2011) have pointed out that the principle of organ balance was discussed and considered in plant morphology by authors such as Johann Wolfgang von Goethe, and Pierre Jean François Turpin and Augustin Pyramus de Candolle.

14 Alphonse de Candolle to Darwin, 14 January 1873, Darwin Correspondence Project, “Letter no. 8737,” accessed on 21 February 2024, https://www.darwinproject.ac.uk/letter/?docId=letters/DCP-LETT-8737.xml. Also published in The Correspondence of Charles Darwin, vol. 21.

15 Darwin to de Candolle, 31 January 1863, Darwin Correspondence Project, “Letter no. 3957,” accessed on 21 February 2024, https://www.darwinproject.ac.uk/letter/?docId=letters/DCP-LETT-3957.xml. Also published in The Correspondence of Charles Darwin, vol. 11.
